# Epigenetic regulation of inflammation by dopamine in primary human macrophages

**DOI:** 10.3389/fimmu.2026.1779072

**Published:** 2026-06-26

**Authors:** Yash Agarwal, Margish Ramani, Samyuktha Manikandan, Kimberly Bonar, John Montilla Luna, Peter J. Gaskill, Stephanie M. Matt

**Affiliations:** 1 Department of Pharmacology and Physiology, Drexel University College of Medicine, Philadelphia, PA, United States; 2 Department of Medicine, Drexel University College of Medicine, Philadelphia, PA, United States

**Keywords:** DNA methylation, dopamine, epigenetics, inflammation, macrophage

## Abstract

**Introduction:**

While dopamine is a monoamine neurotransmitter best known for its roles in reward, motivation, and motor function in the central nervous system, its actions extend beyond neurons and can influence non-neuronal cells via epigenetic mechanisms. An increasing body of literature demonstrates that dopamine signaling is important in immune cells, which express dopamine receptors (DRD1-DRD5) as well as the molecular machinery for dopamine synthesis and metabolism. Dopamine can regulate inflammatory activity, cell trafficking, and disease pathology, yet the epigenetic mechanisms underlying these effects remain poorly understood.

**Methods:**

Primary human monocyte-derived macrophages were treated with dopamine, and DNA methylation at the IL-1β proximal promoter was evaluated alongside IL-1β and epigenetic enzyme gene expression. Associations between donor characteristics, dopamine receptor expression, and dopamine-induced epigenetic responses were also examined.

**Results:**

Dopamine increased DNA methylation at the IL-1β proximal promoter in a DNMT-dependent manner while concurrently increasing IL-1β gene expression. Dopamine treatment also upregulated the expression of several key epigenetic regulators, including TET2, HDAC2, and HDAC6, suggesting coordinated regulation of both DNA methylation and histone modifications that shape inflammatory transcription. Furthermore, baseline dopamine receptor expression and donor demographics, including sex and age, influence the magnitude of these epigenetic responses, highlighting inter-individual variability in macrophage sensitivity to dopaminergic signaling.

**Discussion:**

These findings establish dopamine as a modulator of macrophage inflammation via epigenetic remodeling and provide a mechanistic framework for understanding how peripheral immune cells respond to dopaminergic cues. By linking dopamine signaling, epigenetic regulation, and innate immunity, this work identifies potential targets for therapeutic intervention and supports the use of accessible human immune cells to investigate dopaminergic dysregulation in neuroimmunological disorders.

## 1 Introduction

Dopamine is a monoamine neurotransmitter classically associated with reward, motivation, and motor control in the central nervous system (CNS), signaling through five G protein–coupled receptors (DRD1–DRD5) and tightly regulated by the dopamine transporter (DAT). Beyond these canonical mechanisms, dopaminergic activity interfaces with epigenetic pathways that regulate gene expression independently of DNA sequence. These pathways include DNA methylation, post-translational histone modifications, and non-coding RNA mechanisms, which are mediated by DNA methyltransferases, histone-modifying enzymes, and RNAi machinery, respectively. Through engagement of these epigenetic mechanisms, transient dopamine signals can be translated into stable and enduring changes in transcriptional programs that shape cellular function and behavior over time (1, 2).

Dopamine signaling and epigenetic regulation have a complex, bidirectional interplay. On one hand, dopamine’s canonical actions are shaped by epigenetic mechanisms that regulate the expression of genes involved in dopamine synthesis, signaling, and receptor/transporter function, thereby modulating dopamine availability and influencing cellular responsiveness to dopaminergic cues (3–7) (**Table 1**). Given dopamine’s salient role in neurological disease, altered dopaminergic tone can drive persistent epigenetic changes that underlie long-term transcriptional reprogramming in neuropsychiatric and neurodegenerative disorders, including substance use disorders, depression, schizophrenia, and Parkinson’s disease (24–28) (**Tables 1**–**3**). Conversely, environmentally or pharmacologically induced changes in dopamine levels can themselves trigger epigenetic modifications, including alterations in DNA methylation and histone marks that reshape chromatin accessibility and downstream gene expression (32, 36–38) (**Table 2**). In addition to these indirect mechanisms, dopamine can directly modify histones through dopaminylation (30), adding another layer of regulation through which dopaminergic signaling shapes cellular plasticity. Collectively, these findings position epigenetic regulation as a critical mediator of dopamine-dependent cellular function and dysfunction.

**Table 1:** Epigenetic regulation of dopaminergic signaling.

Species & cell type/region	Disease	Receptor, protein	Experimental design	Method(s) of detection	Finding	Reference
Human SH-SY5Y cells Mouse Striatum, Substantia nigra	Parkinson’s Disease	HDAC6, TH	6-OHDA (100uM or 9ug) Tubastatin A (TBA; 15uM or 25mg/kg)	IHC, qPCR, Western blot, HPLC, Cell viability assays	TBA protects from 6-OHDA-induced cell death and ↓ NLRP3 activation TBA ↓ 6-OHDA-indced NLRP3 activation, astrocyte/microglial activation, and dopaminergic neurotoxicity (TH and dopamine levels)	(8)
Human SK-N-MC cells Mouse Striatum	Toxoplasmosis	miR-132, DRD1, DRD5	*Toxoplasma gondii* infection	Microarray, qPCR, Western blot, HPLC	Toxoplasma ↑ human miR-132 Toxoplasma ↑ mouse miR-132 which was associated with dopamine signaling pathway genes, ↓ DRD1, DRD5, MAOA, DARPP-32 phosphorylation, and ↑ levels of dopamine and its metabolites	(9)
Mouse Hippocampus	Chronic Inflammation	DNMTs, TETs, BH4	LPS (0.33mg/kg)	HPLC, qPCR	LPS ↑ dopamine levels, BH4 synthesis genes, DNMTs, TET2, and ↓ TET1, TET3 and DNA methylation of BH4 synthesis genes	(10)
Mouse Medial Prefrontal Cortex	Substance Use Disorders	DRD1, DRD2	SCH23390 (0.05 mg/kg), raclopride (8 mg/kg), METH (1 mg/kg), modafinil (90 mg/kg)	ChIP, qPCR, Western blot	Modafinil ↑ H3ac at Drd1 promoter and ↑ H4ac at Drd2 promoter; METH and modafinil ↑ H3ac at Drd2 promoter; METH ↑ H4ac at Drd1 promoter	(6)
Mouse Ventral striatum, Medium spiny neurons	Major Depressive Disorder	pan-acH3, H3K27me3, HDAC2	Malvidin-3'-O-glucoside (Mal-gluc; 0.5 ug/kg) and DHCA (5 mg/kg)	qChIP	Mal-gluc ↓ HDAC2 and ↑ acetylation of Rac1, but no effect on Rac1 promoter region in MSN-enriched neurons; PBMC exposure to Mal-gluc ↓ IL-6	(11)
Mouse Anterior cingulate cortex, BV2 microglia	Major Depressive Disorder, Allergic Rhinitis	TET2	TET2 knockout; Ovalbumin (OVA; 200ug); Metformin (Met; 250mg/kg)	Western blot, ELISA, Histology	OVA and TET2 deficiency ↑ depression and anxiety behaviors, ↑ NLRP3/IL-1β pathway; Met reduced these effects by ↑ TET2	(12)
Mouse Prefrontal Cortex	Schizophrenia	DRD1, H3K9ac, HDAC5	PCP (10 mg/kg) and post-treatment Haloperidol (HAL; 1.0 mg/kg), clozapine (CLZ; 10 mg/kg), or sodium butyrate (SB; 0.2, 1.0, or 2.0 g/kg), SCH23390 (0.1 mg/kg)	Western blot, RT-PCR	Repeated PCP treatment ↓ nuclear phospho-CaM kinase II, ↑ HDAC5, and ↓ Ac-H3K9; CLZ and SB, but not HAL, ↑ Ac-H3K9, Ac-K14, and P-H3S10 in PCP mice; SCH23390 prevents ↑ H3ac by CLZ in PCP mice	(13)
Mouse Hippocampus	Early Life Adversity	DRD1, Histone H3	Short-term or long-term separation stress (STSS,or LTSS)	ChIP, qPCR, Western blot	STSS ↑ DRD1; SSTS ↓ DARPP-32 and H3 acetylation of DARPP-32 promoter; LTSS ↑ DARPP-32 and did not change DRD1 expression or H3 acetylation.	(14)
Mouse Nucleus accumbens	Early Life Adversity	DRD1, DRD2	Maternal separation and social isolation	qRT-PCR, Western blot, DNA methylation analysis	Maternal separation + social isolation ↑ hypermethylation in 29 of 31 CpG sites of Drd1a promoter region in female mice; female offspring have ↓ D1 receptor mRNA and protein levels	(15)
Mouse Striatum	Prenatal Stress	DRD1, DRD2, DNMT1, DAT, TH	Restraint stress paradigm with pregnant dams, offspring evaluated up to post-natal-day 60	MeDIP, ChIP, Western blot, qRT-PCR	Prenatal stress ↓ DRD2 mRNA and protein; 5-aza-CdR ↑ DRD2 mRNA and protein; DNMT1 binding to D2R promoter higher in prenatal stress mice vs control; No effect on DAT, TH, DRD1	(16)
Mouse Hypothalamus, VTA	Obesity	TH, DAT, DRD1, DRD2	Control vs high-fat diet (HFD)	qPCR	High-fat diet ↑ TH and DAT mRNA levels in hypothalamus and ↓ in VTA, coupled with ↓ TH and DAT methylation in hypothalamus and ↑ in VTA; HFD ↓ DRD1 mRNA levels in VTA, NAc, and PFC but not hypothalamus; HFD ↓ DRD2 mRNA levels only in VTA	(7)
Mouse Midbrain dopaminergic neurons, Striatum	Chronic Inflammatory Pain	JMJD3, Tyrosine Hydroxylase (TH), NURR1, H3K27me3	JMJD3 conditional knockout or pharmacological inhibition (GSK-J4)	Histology, RT-PCR, ChIP-qPCR, HPLC-MS	JMJD3 deficiency ↓ dopamine content in midbrain and striatum, associated with ↓ TH and NURR1, due to ↑ H3K27m3 repressive effect on *Jmjd3* and *Nurr1* promotor regions	(17)
Rat VTA, Nucleus accumbens	Obesity & Maternity	TH, DRD1, DRD2, DAT	Maternal Standard diet vs high-fat diet (HFD); evaluate differences in offspring	qPCR, Promoter CpG analysis	Maternal HFD ↓ TH gene expression in VTA and NAc of offspring; DRD1 mRNA ↑ in VTA and ↓ in NAc of HFD offspring; DRD2 and DAT mRNA ↓ in VTA and NAc of HFD offspring; Maternal HFD ↑ methylation of TH and DAT promotor regions of offspring	(18)
Rat Pituitary gland	Fetal Alcohol Syndrome	DRD2, DNMT1, DNMT3b, MeCP2, HDAC2, HDAC4, G9a	Pregnant dams fed 6.7% v/v ethanol liquid diet, examined pituitary function in female offspring at day 90. Estrogen replacement therapy used to simulate prolactinoma risk	qPCR, Enzyme immunoassay, Western Blot, Bisulfite DNA methylation assay	Prenatal alcohol-exposed female offspring have ↑ pituitary weight, prolactin protein, and plasma prolactin; alcohol-exposed offspring have ↓ DRD2 mRNA and D2R protein in pituitary, and ↑ CpG methylation of Drd2 promoter; Neonatal rats treated with 5-Aza-CdR or trichostatin-A showed restored DRD2 mRNA levels, pituitary weight, and plasma prolactin in adulthood	(19)
Rat Hippocampus, Cerebellum	Schizophrenia	DRD1, DRD2, DRD4, DRD5, DAT, COMT	Olanzapine (2.5mg/kg)	Genome-wide methylation array	Olanzapine ↑ DRD4 and SLC18A2 methylation in cerebellum	(20)
Rat Midbrain, Striatum	Development	DAT, DNMT1, DNMT3a, DNMT3b, HDAC1/2, HDAC3, HDAC5, HDAC8	Brain samples at post-natal day 3, 7, and 56	qPCR, Western Blot	DAT levels increase by post-natal day 56; accompanied by decreases in DNMT1, 3a, 3b and an increase in HDAC 5 and 8	(3)
Rat Nucleus accumbens	Early Life Adversity	DRD2, miR-9	Maternal deprivation, chronic unpredictable stress	qRT-PCR, Western Blot	Early life stress ↓ miR-9 expression, ↑ DRD2 levels, and ↑ depression-like behavior.	(21)
Rat Ventral mesencephalic tissue, cortical astrocytes, C6 glioma cells	Parkinson’s Disease	BDNF GDNF	Valproic acid (VPA, 1.2mM) SB (0.6-1.2mM), trichostatin A (TSA; 50-100nM)	DA Uptake Assay, ICC, qPCR, ChIP	SB and TSA exert neurotrophic and protective effects on DA neurons in neuron-glia cultures; All three HDAC inhibitors ↑ GDNF and BDNF mRNA in primary cortical astrocyte cultures; HDAC inhibitors ↑ promoter activity of GDNF and induce hyperacetylation of GDNF promoter-associated H3	(22)
Rat Modified Huntington PC12 cells	Huntington Disease	DRD1	Cells modified to express GFP-tagged Huntingtin exon 1	circRNA microarray and miRNA/mRNA interaction analysis	DRD1 gene can be targeted by miR-15a-5p and miR-15b-5p and associated with 4 ↓ circRNAs	(23)

**Table 2:** Epigenetic effects of dopamine-modifying drugs.

Species & cell type/region	Disease	Receptor, protein	Experimental design	Method(s) of detection	Finding	Reference
Human Frontal Cortex	HIV, Substance Use Disorders	DNMT1	History of methamphetamine use, HIV seropositivity	RT-PCR, Western blot, Microarray, ELISA	↑ global DNA methylation in HIV+METH group; ↑ DNMT1 gene and protein expression in HIV+METH+ group; Differential DNA methylation on genes related to dopamine metabolism and transport	(29)
Human, Rat VTA Mouse Striatum	Substance Use Disorders	Histone H3	Non-users & cocaine-dependent Cocaine self-administration (Mice, Rats)	Western blot, Electrophysiology, Fast-scanning cyclic voltammetry, RNA sequencing	Cocaine ↑ histone H3 glutamine 5 dopaminylation (H3Q5dop); H3Q5dop reduction during withdrawal reverses cocaine-induced gene expression changes and ↓ cocaine-seeking behavior; Cocaine users have ↓ H3Q5dop in VTA; Total H3Q5dop ↓ in VTA of cocaine-conditioned rats, blocked by H3Q5A vector transduction H3Q5A transduction ↓ spontaneous action potentials and dopamine release	(30)
Mouse Medial Prefrontal Cortex	Substance Use Disorders	H3ac, H4ac, HDAC1, HDAC2	SCH23390 (0.05 mg/kg), raclopride (8 mg/kg), METH (1 mg/kg), modafinil (90 mg/kg)	ChIP, qPCR, Western blot	METH and modafinil ↑ H3ac (reversed by raclopride), ↓ H4ac (reversed by SCH23390) and ↑ HDAC1, HDAC2; Raclopride prevented METH-induced ↓ H4ac; SCH23390 ↑ HDAC1 and 2; Raclopride ↑ H3ac	(6)
Mouse Striatum, Ventral Midbrain	Substance Use Disorders	Histone H3, HDACs, TH, BDNF	SKF82958, (1 mg/kg), +/- SB (200 mg/kg or 25 mg/kg), Cocaine (15 mg/kg)	ChIP, qRT-PCR	SKF82958 ↑ H3pS10acK14 in striatal neurons, attenuated by MK-801 or H89; SFK32958 + SB ↑ TH and BDNF levels, associated with ↓ H3 acetylation; SB ↑ reward enforcement by SKF82958	(31)
Mouse, Rat Striatum	Substance Use Disorders	HDAC4, HDAC5	Cocaine (20 mg/kg)	Western blot	Cocaine ↑ dephosphorylated HDAC5 S279 and nuclear HDAC4; chronic cocaine administration i↑ the phosphorylation and cytoplasmic localization of HDAC4	(32)
Mouse, Rat Striatum	Substance Use Disorders	HDAC5	Cocaine (5 or 20 mg/kg)	ChIP, Western Blot	Cocaine and cAMP ↑ nuclear HDAC5; Cocaine ↓ phosphorylation of HDAC5 via P-S259 and P-S498	(33)
Mouse Striatum (ST), NAc, Hippocampus, Cingulate Cortex (Cg), Amygdala	Psychiatric Disorders	HDAC1, HDAC2, HDAC3, HDAC4, HDAC5, HDAC7, HDAC8, HDAC10	S)-citalopram oxalate (ECM), duloxetine hydrochloride (DLX), and mirtazapine (MIR), sodium valproate (VPA), lithium chloride (Li), lamotrigine (LTG), levetiracetam (LTM), olanzapine (OLZ), clozapine (CLZ), clomipramine (CLM)	Western Blot	Several compounds ↑ AcH3 protein levels in the NAc, while others ↑ HDAC2, HDAC3, and HDAC5 in the ST; Lithium ↓ HDAC1, HDAC3, HDAC4, HDAC5, HDAC7, HDAC8, and HDAC10 in the ST Multiple effects reported	(34)
Mouse Prefrontal Cortex (PFC), Striatum	Schizophrenia	H3K9me2, H3K9me3, H3K4me3, H3K27me3, NR4A2, AKT1	WT vs D2-autoreceptor KO mice (DA-D2RKO) Quinpirole (QUIN; 0.2 mg/kg), clozapine (CLZ; 3 mg/kg), or amphetamine (AMPH; 1, 3 mg/kg)	Microarray, Western blot, Microscopy, ChIP	DA-D2RKO mice show ↑ H3K9me2/3 repressor mark in PFC, but not striatum; H3K9me3 enriched in NR4A2 and AKT1 promoters of PFC in DA-D2RKO mice with ↓ in NR4A2 and AKT1 protein; Pre-pulse inhibition is ↓ in DA-D2RKO mice; Chronic QUIN treatment restores H3K9me3 levels in PFC, but CLZ does not	(35)
Mouse Striatum	Parkinson Disease	H3K27, H3K4	L-DOPA treatment of hemiparkinsonian mice	Western blot, Microscopy, ChIP-seq, RNA-seq	L-DOPA ↑ H3K27me3S28 phosphorylation, associated with ↓ Rnf2 binding to de-repress Atf3, Klf4, and Npas4, potentially through D1-like mechanism. Inhibited by MSK-1 knockout, PP1 inhibition, and DARPP-32 T34A mutation	(36)
Rats Prefrontal cortex	Substance Use Disorders	KDM6B, BRD4	WT, heterogyzous DAT mutation, or homozygous DAT knockout Cocaine (10mg/kg)	qRT-PCR	Sex-dependent effects of full and heterozygous DAT knockout associated with changes in KDM6B and BRD4 following cocaine treatment	(37)
Rat Midbrain	Substance Use Disorders	HDAC2, H3K9	morphine sulfate (10mg/kg) CI-994 (20μM)	Western blot, Electrophysiology	Morphine ↑ HDAC2; CI-994 reverses morphine-induced pre- and postsynaptic changes of GABAergic signaling; CI-994 ↑ H3K9ac in midbrain slices from morphine-treated rats	(38)
Rat Astrocytes	Substance Use Disorders	PARP1, CTCF	DA (75 μM), SCH23390 (50μM), sulpiride (20μM), 2-APB (50μM)	Microscopy, RNA seq, qRT-PCR,	D1-like and D2-like activity are involved in DA-mediated changes in astrocytic processes; DA ↑ Nr4a3 and ↓ Klf4 in hippocampal slices	(39)

**Table 3:** Dopamine-associated regulation of epigenetics in non-neuronal cells.

Species & cell type/region	Disease	Receptor, protein	Experimental design	Method(s) of detection	Finding	Reference
Human THP-1 macrophages	Substance Use Disorders	H3K4, H3K27, CCR5	DA (1μM), Meth (60μM), or HIV Tat (10ng/mL)	CHIP-qPCR, Flow Cytometry	DA ↑ H3K4me3 and H3K27Ac at CCR5 promotor, but Meth does not; DA ↑ CCR5 expression transiently, but SKF38393 ↓ CCR5	(40)
Human Peripheral blood mononuclear cells	Substance Use Disorders	DNMTs, TETs	Amphetamine (0.3ug/mL), Cocaine (3ug/mL), Decitabine (0.3uM)	qPCR, DNMT and TET activity assays, ELISA, Next-generation sequencing	Stimulants ↑ DNMT1, C TET1–3 mRNA and TET activity, hypermethylated IL-10, and hypomethylated ATP2B4, and ↓ global 5-hmC levels; decitabine blocked cocaine’s effects on TETs, DNMTs, and cytokines	(41)
Human Peripheral blood, Nucleus accumbens	Alcohol Use Disorder	DAT1	Alcohol-free & alcohol-dependent	Methylation-Specific PCR, fMRI	Decreased methylation of SLC6A3 promoter region correlated with nucleus accumbens activation on fMRI; correlation lost in alcohol-dependent individuals	(27)
Human Peripheral blood	Cannabis Use Disorder	DAT1	Cannabis use disorder diagnosis and healthy control participants	Methylation-Specific PCR	↓ DAT1 methylation significant predictor of cannabis use disorder	(42)
Human Monocytes, T cells	HIV	NR4A2, DRD3	HIV diagnosis with cognitive impairment	Genome-wide DNA methylation, RNA sequencing	HIV infection differentially methylates CpG sites in 416 genes in monocytes, 736 genes in T-cells, and 81 genes overlapping in both cell types, from CI and non-CI patients; Altered CpG methylation index for DRD3 gene in cognitively impaired cohort	(43)
Human CD14+CD16+ Monocytes	PHEO	beta-adrenoreceptor, H3K4me3	Pheochromocytoma/paraganglioma (PHEO) diagnosis, (Nor)adrenaline (10nM) +/- LPS (10ng/mL)	RNA-seq, ChIP	Pheochromocytoma/paraganglioma ↑ H3K4me3, which promotes expression of TNFA, IL1B, CXCL2, CXCL3 and IL8	(44)
Human Peripheral blood, Striatum	ADHD	DAT1	Attention deficit hyperactivity disorder (ADHD) diagnosis	Methylation-Specific PCR, Quantitative bisulfite pyrosequencing	Inverse correlation between DAT promoter methylation and DAT availability in nucleus in people with ADHD	(45)
Human Peripheral blood mononuclear cells	Parkinson Disease	DAT1	Parkinson disease diagnosis	Methylation-Specific PCR, Quantitative bisulfite pyrosequencing	CpG sites for DAT promoter may be methylated in high-severity PD patients relative to low severity patients	(28)
Human White blood cells	General Intelligence	DRD2	Control participants	Illumina DNA Methylation analysis	DRD2 methylation in white blood cells is inversely correlated with general intelligence score	(46)
Human Buccal cells	Cognition	DRD4, COMT, DBH, DAT, DRD1, DRD2	Monozygotic Twin Study	Bisulfite DNA Methylation Array, Digit span and flanker task assessments	Twin differences in DRD4 methylation predicted differences in short-term memory; Twin differences in COMT, DBH, DAT, DRD1, DRD2 methylation predicted differences in response inhibition	(47)
Human Tumor Biopsy & Pediatric CNS tumor lines	Pediatric brain cancer	DRD4	5-Aza-CdR (5 μM)	MTT assay, Flow cytometry, Bisulfite PCR, qPCR, ChIP-qPCR, ELISA, Western blot	Genome-wide methylation analysis showed DRD4 as a tumor-specific candidate, supported by DRD4 methylation analysis of tumor cell lines; 5-aza-2’-deoxycytidine (5-Aza-CdR) ↓ tumor viability and ↑ DRD4 expression, but methylation of DRD4 unchanged; 5-Aza-CdR ↓ H3K27me3 in DRD4 promoter; EZH2 enriched in DRD4 promoter; shRNA for EZH2 ↑ DRD4 mRNA and protein; D4R agonist P233 ↑ ERK1/2 levels; shRNA for EZH2 ↑ ERK1/2	(48)
Chimpanzee Peripheral blood	Social behavior	DRD2	N/A	Personality questionnaire, CpG methylation array	DRD2 CpG methylation strongly associated with extraversion	(49)
Mouse Testicular germ cells	Substance Use Disorders	DRD1, H3K9me3, H3K27me3, H4K16ac, HDAC1/2, SIRT1, KAT8/MOF	Cocaine (10mg/kg) +/- SCH23390 (0.5mg/kg) pre-treatment	Western blot, RT-PCR	Cocaine ↓ DRD1 mRNA and protein, reverted by SCH23390; Cocaine ↑ H3K9me3, H3K27me3, H3K9ac, and H4K16ac, reverted by SCH23390; Cocaine ↓ HDAC1 and HDAC2 but ↑ SIRT1, KAT8/MOF, reverted by SCH23390; Cocaine and SCH23390 ↓ KDM1A; Cocaine and SCH23390 ↑ KMT1C	(50)
Mouse Nucleus accumbens (NAc), Cerebellum (Cer) Peripheral blood mononuclear cells (PBMCs)	Substance Use Disorders	DNMTs TETs	Cocaine (15 mg/kg)	DNMT and TET activity assays qPCR LC-ESI-MS/MS	30 min cocaine ↓ DNMT1, DNMT3a, TET1, and TET2 mRNA and 24h cocaine ↑ DNMT mRNA and enzyme levels in NAc and PBMCs; Cocaine withdrawal ↑ DNMTs in NAc, Cer, and PBMCs and ↓ TET1 and TET3 in NAc; cocaine withdrawal ↑ DNMT and ↓ TET levels associated with changes in 5-mC and 5-hmC	(51)
Rat Liver	Schizophrenia	DRD1, DRD2, DRD4, DRD5, DAT, COMT	Olanzapine (2.5mg/kg)	Genome-wide methylation array	Olanzapine differentially methylated DRD1, DRD2, DRD5 in liver and ↓ COMT methylation	(20)

While dopamine-mediated epigenetic regulation has been studied primarily in the context of neuronal dysfunction in neuropsychiatric and neurodegenerative diseases, these disorders are also characterized by chronic inflammation (52–54). Interactions between dopaminergic signaling and epigenetic regulation occurs across multiple cell types, suggesting substantial crosstalk between peripheral immune regulation and brain function (27, 28, 40, 41) (**Table 3**). Human studies of epigenetic modifications of dopaminergic machinery in peripheral blood have reported associations with cognitive performance and psychiatric symptom severity (42, 47, 55), and inflammatory stimuli, including LPS or infections such as toxoplasmosis or HIV (9, 10, 29, 43), can induce epigenetic changes that impact dopamine signaling and metabolism. Recently, deficiency of the histone demethylase JMJD3 (KDM6B) was shown to disrupt dopamine biosynthesis in the midbrain and exacerbate chronic inflammatory pain (17), providing direct evidence that epigenetic regulation of dopaminergic pathways can influence inflammatory outcomes. However, existing studies have largely focused on epigenetic regulation of dopaminergic machinery rather than inflammatory gene targets in immune cells, highlighting a critical knowledge gap.

This expanding understanding of dopamine’s involvement in both the brain and the immune system points to a broader role in regulating inflammation throughout the body. Dopamine signaling is not restricted to neuronal cells; it is also present in peripheral tissues, where various immune cell populations express the molecular machinery necessary for dopamine synthesis, metabolism, and signaling. This enables direct dopaminergic regulation of immune function (52, 56–58). Consistent with this, both endogenous dopamine and dopaminergic drugs have been shown to modulate immune cell activation and inflammatory responses across diverse immunological contexts. Several groups, including our own, have documented dopamine receptor expression and DAT in human myeloid cells (59–66), supporting that these cells can respond to dopamine. Specifically, we found that dopamine can exert an inflammatory effect on myeloid cells such as primary human monocyte-derived macrophages (MDM) and iPSC-derived microglia, in part by upregulating the NF-kB pathway, increasing NLRP3 priming and IL-1β expression/release (67, 68). This has been shown to be mediated by levels of dopamine receptor expression, in particular the D1-like receptors (DRD1 and DRD5) (68). However, it is not known if dopamine modulates macrophage immune responses through mechanisms that involve epigenetic regulation.

Macrophages are central regulators of innate immune responses and exhibit significant functional plasticity in response to environmental cues. Given their post-mitotic nature and longevity, macrophages face the challenge of maintaining genomic stability while remaining highly adaptable. Epigenetic mechanisms regulate this plasticity by controlling chromatin accessibility at inflammatory gene loci, which in turn facilitates the binding of key transcription factors, such as NF-κB, STAT, and MAPK (69, 70). This binding is crucial for determining the strength and duration of inflammatory responses in macrophages. In particular, the epigenetic control of genes like IL-1β, a critical mediator of inflammation, plays a pivotal role in the macrophage’s ability to mount an effective immune response. While many studies across various cell types, including macrophages, have examined changes in IL-1β DNA methylation (71–74), no research has specifically explored the impact of dopamine on IL-1β DNA methylation or other epigenetic modifications in macrophages. Given that macrophages can often be exposed to high dopamine levels and share conserved epigenetic regulatory machinery with neural cells, they represent a biologically relevant and tractable system for studying dopamine-driven epigenetic remodeling. In this study, we investigate the role of epigenetic mechanisms in dopamine-mediated regulation of inflammation in primary human monocyte-derived macrophages. Our findings demonstrate that dopamine increases DNA methylation at the IL-1β proximal promoter in a DNA methyltransferase–dependent manner and increases the expression of key epigenetic regulators, including TET2, HDAC2, and HDAC6. Further, we show that donor demographics and dopamine receptor expression influence these epigenetic changes. Collectively, these findings establish a mechanistic link between dopamine signaling, epigenetic regulation, and macrophage inflammation, providing new insights into how dopaminergic modulation of innate immune responses could contribute to disease pathogenesis.

## 2 Methods

### 2.1 Reagents

RPMI 1640 medium (11875119, Gibco), Fetal Bovine Serum (FBS; MT35010CV, Gibco), Human AB serum (100-512-100, Gemini Bio), HEPES (BP2991), Penicillin/Streptomycin (15140163, Gibco), and sterile dH2O (10977023, Invitrogen) were all purchased from ThermoFisher. Macrophage-colony stimulating factor (M-CSF; 315-02) was obtained from Peprotech. Dopamine hydrochloride (DA; H8502), 5-deoxyazacytidine (dAZA; A3656) and 5-azacytidine (AZA; A2385) were obtained from Sigma-Aldrich. TaqMan Fast Universal Master Mix, and PCR assay probes for DRD1-5 (Hs00265245_s1, Hs00241436_m1, Hs00364455_m1, Hs00609526_m1, Hs00361234_s1), IL1B (Hs01555410_m1), DNMT1 (Hs00945875_m1), DNMT3A (Hs01027166_m1), DNMT3B (Hs00171876_m1), TET2 (Hs00325999_m1), HDAC2 (Hs00231032_m1), HDAC6 (Hs00997427_m1), KDM6B (Hs00996325_g1), KAT2A (Hs00904943_gH), and 18S (4319413E) genes were purchased from Applied Biosystems. Reagents were stored and handled according to manufacturer’s conditions.

Macrophage medium was composed of RPMI 1640 supplemented with 10% heat inactivated FBS, 5% human AB serum, 1% HEPES, 1% Penicillin/Streptomycin, 10ng/mL MCSF. M-CSF lyophilized powder was re-suspended in sterile dH2O at a concentration of 100ug/mL and added to fresh culture medium immediately before use. Dopamine hydrochloride (DA) was resuspended in sterile dH_2_O in the dark at a stock concentration of 10 mM that was then aliquoted and frozen for two months or until use, whichever occurs first. New aliquots were prepared from lyophilized powder every two months. All dopamine preparations and treatments were performed in the dark using 10^-6^M dopamine, with dopamine treatment occurring immediately after thawing the dopamine. dAZA lyophilized powder was resuspended in dH2O to a stock concentration of 10mM and stored at -20C until use. AZA was prepared in DMSO at a stock concentration of 10mM. On the day of the experiment, a fresh vial was thawed and added 1:1000 to culture medium for a final concentration of 1μM.

### 2.2 Cell isolation and culture

Human peripheral blood mononuclear cells (PBMC) were separated from blood obtained from de-identified healthy donors from the New York Blood Center (Long Island City, NY, USA), University of Pennsylvania Human Immunology Core (Philadelphia, PA, USA), and BioIVT (Westbury, NY, USA). Live PBMC counts were estimated using the Nexelcom Cellometer Auto 2000 (Revvity), and 10 million PBMC were used to determine the proportion of monocytes in each donor. Monocyte isolation was performed using the Pan Monocyte Isolation Kit (Miltenyi Biotec, 130-096-537), and the isolated monocytes were counted. According to this result, 950,000 monocytes per well were plated in 6-well plates (Corning) in filter-sterilized macrophage medium (RPMI-1640 supplemented with 10% FBS, 5% human AB serum, 10 mM HEPES, 1% P/S, and 10ng/mL M-CSF). Wells were washed once in plain RPMI medium at three days *in vitro* and replaced with macrophage medium to remove non-adherent PBMCs. Adherent cells matured into monocyte-derived macrophages (MDM) by 6–7 days in culture, as previously described (59, 64, 67). Limited, de-identified demographic information (age, gender, ethnicity, blood type and cytomegalovirus (CMV) status) was obtained, although all data categories were not available for each donor. All donors (N = 53) were included in analyses of dopamine receptor expression, but incomplete demographic information limited inclusion in some correlation analyses.

### 2.3 Drug treatments

For dopamine treatments without DNMT inhibitors, mature MDM were treated on day 7 with 1μM dopamine for 3 hours and cell contents were immediately collected in RNA/DNA Lysis Buffer (Zymo Research). For DNMT inhibition experiments, MDM were treated on day 6 (t=0h) with either vehicle (sterile dH2O; 1:1000), 1μM of 5- azacytidine (AZA), or 5-deoxyazatycidine (dAZA) in fresh macrophage medium. After 24h (t=24h), medium was replaced with a second 1μM treatment with vehicle, 1μM 5-AZA, or 1μM 5-dAZA in fresh macrophage medium. After 21h (t=45h from start of treatments), cells were treated with either vehicle or 1μM dopamine for 3 hours. Cell contents were harvested using RNA/DNA Lysis Buffer in the Quick DNA/RNA Miniprep Kit.

### 2.4 Quantitative RT-PCR

Total RNA was extracted from cells using either Trizol (15596026, Invitrogen) or Quick DNA/RNA Miniprep Kit (D7001, Zymo Research), and RNA quantity and purity were determined with the NanoDropOne spectrophotometer (Nanodrop Technologies). RNA (1μg per sample) was used to synthesize cDNA from each donor using the High-Capacity Reverse Transcriptase cDNA Synthesis Kit (4368814, Applied Biosystems). 12.5ng cDNA was used per well, with each gene run in triplicate wells for technical control. TaqMan Universal PCR Master Mix (4304437, Applied Biosystems) was used for the PCR reaction according to manufacturer’s instructions. All dopamine receptor subtypes, epigenetic enzyme genes, and the housekeeping gene 18S were amplified from cDNA by quantitative PCR (qPCR) on a QuantStudio 7 (Applied Biosystems) using gene-specific primers (see reagents section above for primer information).

### 2.5 Methylation-specific quantitative RT-PCR

Total genomic DNA (gDNA) was extracted using either Trizol (15596026, Invitrogen) or the Quick DNA/RNA Miniprep Kit (D7001, Zymo Research) according to manufacturer’s instructions. gDNA concentration and purity was measured using the NanoDropOne spectrophotometer and was bisulfite-treated using the EZ DNA Methylation Kit (D5001, Zymo Research) according to manufacturer’s instructions. Methylation status of IL1B was assessed via methylation-specific real-time PCR (MSP) using custom primers designed with Methprimer software (http://www.urogene.org/methprimer/) (75). Product specificity was determined by melt curve analysis and gel electrophoresis. Primer sets targeted a methylated and unmethylated CG dinucleotide in DNA associated with the promoter region of IL1B (**Table 4**). Unmethylated beta-actin was used as a reference gene as previously published (76). Reactions were run using 25ng/mL genomic DNA, 100nM forward and reverse primer per reaction, and iTaq Universal SYBR Green Master Mix (1725121, Bio-Rad), and qPCR was run on a QuantStudio 7 with melt curve analysis for each run. Both high-methylated and low-methylated human genomic DNA (80-8061-HGHM5 and 80-8062-HGUM5 from EpigenDx) were bisulfite treated and used as controls for the primer sets. The high-methylated control amplified for the methylated primer and the low-methylated control amplified for the unmethylated primer. However, amplification for MDMs for the unmethylated primer was not detectable in the majority of samples. This suggests this region is predominantly methylated, and we focused on the methylated primer set.

**Table 4:** Primers used in MSP experiments.

Target gene	Primer sequence (5'-3')
Human IL-1β Methylated	ATATATTAAATTTAAATATTTTTTTAACGT
	TACAAATATACATTATTTTCCTAACAATCG
Human IL-1β Unmethylated	TATATATTAAATTTAAATATTTTTTTAATG
	ACAAATATACATTATTTTCCTAACAATCAT
Human Beta –actin	TGGTGATGGAGGAGGTTTAGTAAGT
	AACCAATAAAACCTACTCCTCCCTTAA

### 2.6 Statistics

Prior to analysis, all data were normalized to the mean of the vehicle treated condition. Statistical analysis of gene expression data was performed on data normalized to 2^-ΔCT^. To determine the appropriate statistical tests, data sets were evaluated by analysis of skewness and evaluation of normality and lognormality to determine the distribution of the data. Extreme data points presumed to be technical outliers were identified via ROUT test (Q = 0.1%) and removed from analysis. *Post-hoc* analyses were performed when appropriate. All data analysis was performed using GraphPad Prism 10.2 (Graphpad, La Jolla, CA). p < 0.05 was considered significant.

## 3 Results

### 3.1 DNMT inhibition blocks dopamine-induced expression of IL-1β and dopamine increases IL-1β DNA methylation in primary human macrophages

**Figure 1:**
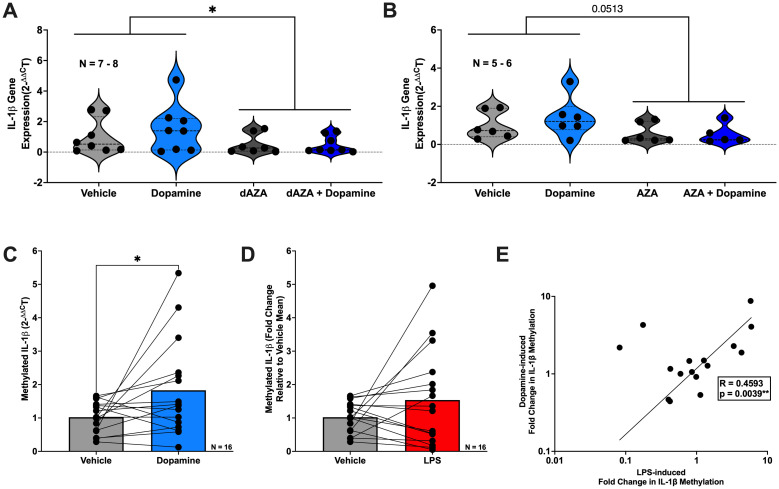
DNMT inhibition blocks dopamine-induced expression of IL-1β and dopamine increases IL-1β DNA methylation in primary human macrophages. Primary human monocyte-derived macrophages (hMDM) were treated with dopamine (10^-6^M) for 3 hours with or without pre-treatment using hypomethylating agents 5-aza-2’-deoxycytidine (dAZA; 10^-6^M), 5-azacytidine (AZA; 10^-6^M), or vehicle control. **(A)** Dopamine increased IL-1β mRNA expression, an effect inhibited by dAZA (n= 7-8, *p<0.05). **(B)** AZA pretreatment produced a similar inhibitory effect on dopamine-induced IL-1β expression, trending toward statistical significance (n=5-6, p=0.0513). **(C)** Dopamine treatment significantly increased methylated IL-1β levels compared to vehicle control (n=16, p<0.05). **(D)** Lipopolysaccharide (LPS) increased IL-1β DNA methylation; however, this effect did not reach statistical significance (n=16). **(E)** LPS-induced changes in IL-1β DNA methylation were significantly correlated with dopamine-induced changes within the same donors, indicating consistent donor-specific responsiveness across stimuli (n=16, **p<0.01).

Given the central role of DNA methylation in controlling both signaling of dopamine and inflammation, we investigated whether DNA methylation contributes to dopamine-mediated regulation of IL-1β in primary human monocyte-derived macrophages (MDM). To investigate whether dopamine’s effects on IL-1β in MDM is mediated by DNA methylation, we used two DNMT inhibitors, 5-deoxyazacytidineand (dAZA) and 5-azacytidine (AZA). MDM were pretreated with either DNMT inhibitor (1μM) for 48 hours and then exposed to dopamine for 3 hours at 1μM, which represents a physiologic concentration of dopamine in many tissues throughout the body (56). The 3-hour timepoint was used as we have previously demonstrated dopamine’s effects on inflammation during this window (67, 68). Cells were then collected for assessment of IL1B mRNA. We previously showed that dopamine-mediated changes in IL1B mRNA are correlated at the protein level using donor-matched assays from independent donor cohorts (67, 68). Thus, we assessed mRNA here to specifically evaluate the effects of dopamine on transcriptional regulation of IL-1β. Pretreatment with dAZA or AZA blocked dopamine’s effects on IL1B (**Figure 1A**; rm 2-way mixed effects model and *post-hoc* with Tukey’s multiple comparisons, dAZA, *p = 0.0260 F (1,7) = 7.913; **Figure 1B**, rm 2-way mixed effects model and *post-hoc* with Tukey’s multiple comparisons, AZA, p = 0.0513 F (1,5) = 6.5), suggesting DNA methylation may play a role in dopamine’s inflammatory effects.

We then wanted to assess whether specific DNA methylation of IL-1β was affected by dopamine. MDM were treated with 1μM dopamine for 3 hours, and DNA was isolated, bisulfite treated, and examined for IL1B DNA methylation at the proximal promoter, as others have done similarly (71, 77, 78). Dopamine significantly increased IL1B DNA methylation (**Figure 1C**, Vehicle vs Dopamine, n = 16, Wilcoxon test, *p = 0.0335, sum of (+,-) ranks 109, -27), providing a mechanistic basis for the observation that DNMT inhibition blocked dopamine-induced increases in IL1B transcription.

Given that lipopolysaccharide (LPS) is a well-established inducer of IL-1β and is known to influence DNA methylation during myeloid inflammatory responses (71, 79–81), we assessed whether LPS (10ng/mL) altered DNA methylation at the IL1B promoter in a manner comparable to dopamine. Although the increase in DNA methylation with LPS did not reach statistical significance (**Figure 1D**, Vehicle vs LPS, n = 16, paired t-test, p = 0.1759, t = 1.421, df = 15), LPS- and dopamine-associated methylation changes in the same MDM donors were positively correlated (**Figure 1E**, n = 16, Pearson r = 0.6777, **p = 0.0039), consistent with the possibility that these stimuli may converge on similar regulatory mechanisms.

### 3.2 Dopamine increases expression of epigenetic enzymes in primary human macrophages

**Figure 2:**
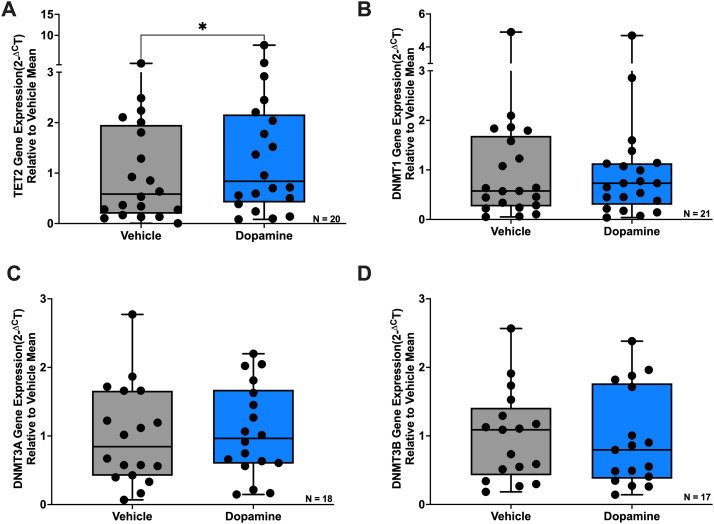
Dopamine selectively increases TET2 expression without altering DNMT expression in primary human macrophages. Primary human monocyte-derived macrophages (hMDM) were treated with dopamine (10^-6^M) for 3 hours. **(A)** Dopamine significantly increased TET2 mRNA expression (n=20, *p<0.05). **(B)** DNMT1 mRNA expression was not significantly altered by dopamine treatment (n=21). **(C)** DNMT3A mRNA expression was not significantly altered by dopamine treatment(n=18). **(D)** DNMT3B mRNA expression was not significantly altered by dopamine treatment (n=17).

Given our observation that dopamine increased DNA methylation at the IL1B proximal promoter in a DNMT–dependent manner, we next examined whether dopamine more broadly influences the expression of enzymes that regulate DNA methylation in MDM. We focused on DNMT1, which is primarily responsible for maintenance of DNA methylation patterns during cell division, and DNMT3A/DNMT3B, which mediate *de novo* DNA methylation, and have all been implicated in regulation of inflammatory gene expression (82–84). We also examined TET2, a key enzyme involved in active DNA demethylation and resolution of macrophage inflammatory responses (12, 85–88). MDM were treated with 1μM dopamine for 3 hours, and dopamine increased TET2 mRNA (**Figure 2A**, Vehicle vs Dopamine, n = 20, Wilcoxon test, *p = 0.0400, sum of (+,-) ranks 160, -50) while DNMT1, DNMT3A, and DNMT3B mRNA were unchanged (**Figures 2B–D**). This indicates at a global level that dopaminergic signaling may modulate enzymes involved in active DNA demethylation.

**Figure 3:**
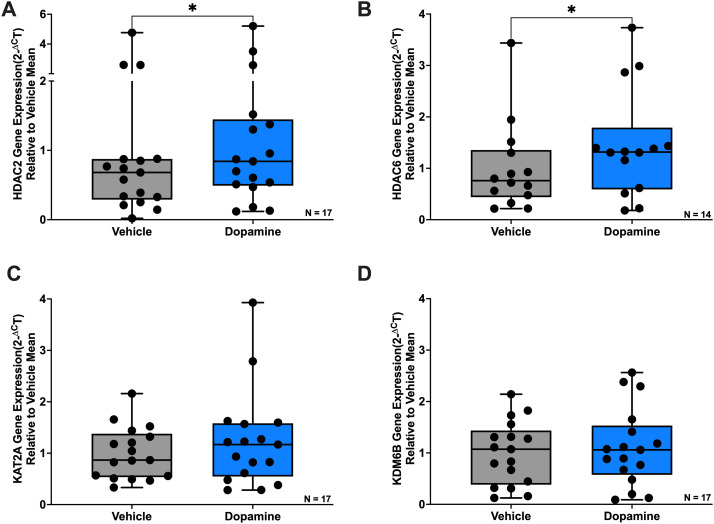
Dopamine increases HDAC2 and HDAC6 expression in primary human macrophages. Primary human monocyte-derived macrophages (hMDM) were treated with dopamine (10^-6^M) for 3 hours **(A)** Dopamine significantly increased HDAC2 mRNA expression (n=17, *p<0.05). **(B)** Dopamine significantly increased HDAC6 mRNA expression (n=14, *p<0.05). **(C)** KAT2A mRNA expression was not significantly altered by dopamine treatment (n=17). **(D)** KDM6B mRNA expression was not significantly altered by dopamine treatment (n=17).

In addition to regulating DNA methylation, epigenetic control of inflammatory gene expression in macrophages is mediated by histone modifications, including histone acetylation and methylation, that shape chromatin accessibility (70). Given our findings that dopamine modulates enzymes involved in DNA methylation, we next examined whether dopamine also influences the mRNA expression of epigenetic regulators involved in histone modification in MDM. We focused on HDAC2 and HDAC6 due to their established roles in regulating behaviors associated with dopaminergic signaling and substance use disorders (8, 89–91) as well as inflammation in macrophages through histone deacetylation (92, 93). We also examined KAT2A, a histone acetyltransferase that promotes inflammation by increasing histone acetylation (94), and KDM6B, a histone demethylase that removes repressive H3K27me3 marks and is involved in macrophage inflammatory signaling (95, 96). MDM were treated with 1μM dopamine for 3 hours, and dopamine increased HDAC2 mRNA (**Figure 3A**, Vehicle vs Dopamine, n = 17, Wilcoxon test, *p = 0.0267, sum of (+,-) ranks 123, -30) and HDAC6 mRNA (**Figure 3B**, Vehicle vs Dopamine, n = 14, Wilcoxon test, *p = 0.0353, sum of (+,-) ranks 86, -19), while KAT2A and KDM6B mRNA were unchanged. These results suggest that dopamine’s influence on inflammatory gene expression is not limited to DNA methylation but also extends to histone modifications.

### 3.3 Sex and age influence dopamine-induced changes in epigenetic enzymes in human macrophages

Having established that dopamine modulates IL-1β DNA methylation and the expression of enzymes involved in DNA methylation and histone modifications in macrophages (MDM), we next considered the known influence of biological sex and age on both dopaminergic signaling and immune responses. We therefore evaluated whether these demographic factors contributed to variability in dopamine-mediated epigenetic regulation. Our analyses revealed that the magnitude of dopamine-induced epigenetic changes varied by sex (males versus females) and age (donors under 40 versus over 40). While no significant differences were observed in IL-1β DNA methylation (data not shown), males exhibited a 2.78-fold increase in DNA methylation, compared to a 1.26-fold increase in females. Similarly, donors under 40 had a 2.34-fold increase, while donors over 40 showed a 1.52-fold increase.

**Figure 4:**
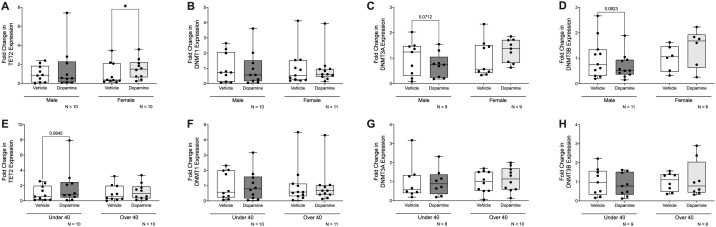
Sex and age influence dopamine-associated regulation of enzymes involved in DNA methylation in primary human macrophages. Data from **Figure 2** were stratified by donor sex and age to assess potential sources of inter-donor variability. **(A)** Dopamine significantly increased TET2 mRNA expression in hMDM from female donors but not male donors (n=10 per group, *p<0.05). **(B)** Dopamine did not significantly alter DNMT1 gene expression in either male (n=10) or female (n=11) hMDM. **(C)** Dopamine treatment showed a decreasing trend in DNMT3A expression in male hMDM (n=9; p=0.0712) and a non-significant increase in female hMDM (n=9). **(D)** Dopamine treatment showed a decreasing trend in DNMT3B expression in male hMDM (n=11; p=0.0823) and a non-significant increase in female hMDM (n=6). **(E)** Dopamine treatment showed an increasing trend in TET2 gene expression in donors under 40 years of age (n=10, p=0.0645), but not donors aged 40 years or older (n=10). **(F–H)** No age-associated differences were observed for dopamine-induced changes in **(F)** DNMT1, **(G)** DNMT3A, or **(H)** DNMT3B expression.

For TET2, dopamine significantly increased its expression in males, but this effect was not observed in females (**Figure 4A**, Males Vehicle vs Dopamine, n = 10, Wilcoxon test, *p = 0.0273, sum of (+,-) ranks 49, -6). Interestingly, for DNMT3A and DNMT3B, while overall dopamine did not significantly alter their expression, there were trends suggesting dopamine decreased their expression in males (**Figure 4C**, Males Vehicle vs Dopamine, n = 9, paired t-test, p = 0.0712, t = 2.4079, df = 8; **Figure 4D**, Males Vehicle vs Dopamine, n = 11, paired t-test, p = 0.0823, t = 1.931, df = 10). Regarding age, only a trend toward increased TET2 expression in donors under 40 was observed (**Figure 4E**, Under 40 Vehicle vs Dopamine, n = 10, Wilcoxon test, p = 0.0645, sum of (+,-) ranks 46, -9), with no significant changes in donors over 40.

**Figure 5:**
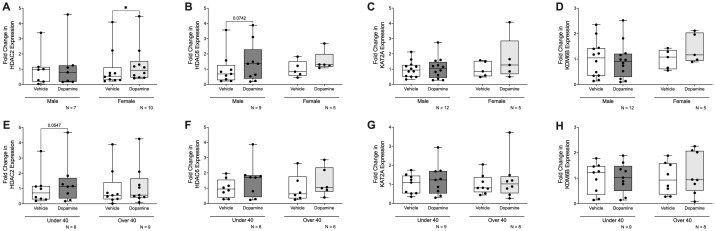
Sex and age influence dopamine-associated regulation of enzymes involved in histone acetylation and methylation in primary human macrophages. Data from **Figure 3** were stratified by donor sex and age to assess potential sources of inter-donor variability. **(A)** Dopamine significantly increased HDAC2 mRNA expression in hMDMs from female donors (n=10, *p<0.05) but not male donors (n=7). **(B)** Dopamine treatment showed an increasing trend in HDAC6 expression in male hMDMs (n=9; p=0.0742) and a non-significant increase in female hMDMs (n=5). Dopamine treatment did not reveal sex-associated differences in **(C)** KAT2A or **(D)** KDM6B gene expression (n=17 donors each). **(E)** Dopamine treatment showed an increasing trend in HDAC2 expression in donors under 40 years of age (n=8, p=0.0547), but not in donors aged 40 years or older (n=9). No age-associated differences were observed for dopamine-induced changes in **(F)** HDAC6 (n=14), **(G)** KAT2A (n=17), or **(H)** KDM6B (n=17) expression.

For HDAC2, dopamine significantly increased its expression in females, but this effect was not observed in males (**Figure 5A**, Females Vehicle vs Dopamine, n = 10, Wilcoxon test, *p = 0.0488, sum of (+,-) ranks 47, -8). In contrast, dopamine showed a trend toward increased HDAC6 expression in males, but this was not observed in females (**Figure 5B**, Males Vehicle vs Dopamine, n = 9, Wilcoxon test, p = 0.0742, sum of (+,-) ranks 38, -7). For age, there was only a trend toward increased HDAC2 expression in donors under 40 (**Figure 5E**, Under 40 Vehicle vs Dopamine, n = 8, Wilcoxon test, p = 0.0547, sum of (+,-) ranks 32, -4), with no significant change in donors over 40.

Together, these findings highlight the potential role of demographic factors in shaping macrophage epigenetic responsiveness to dopamine. Even when no overall change in enzyme expression was observed, demographic breakdowns revealed subtle effects that may have been masked in the aggregate analysis. These results suggest that sex and age should be considered as important factors when studying dopamine’s impact on immune function and epigenetic regulation in macrophages.

### 3.4 Epigenetic enzyme expression predominantly correlates with D1-like dopamine receptor expression in human macrophages

**Figure 6:**
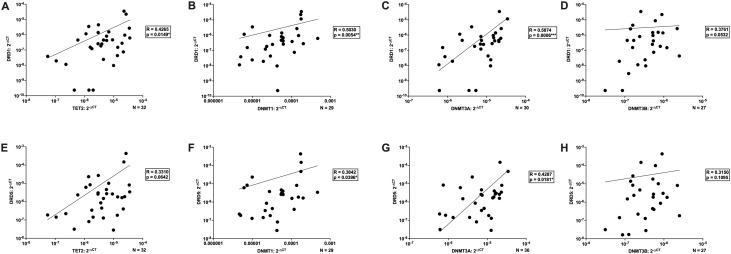
Baseline D1-like receptor expression correlates with enzymes involved in DNA methylation in primary human macrophages. Donors from **Figure 2** were combined with an independent archival cohort to assess mRNA correlations between dopamine receptor expression and epigenetic enzyme expression. DRD1 expression was significantly correlated with **(A)** TET2 (n=32, *p<0.05), **(B)** DNMT1 (n=27, **p<0.01), and **(C)** DNMT3A (n=30, ***p<0.001), but not with **(D)** DNMT3B expression (n=27, p=0.0532). DRD5 expression was not correlated with **(E)** TET2 (n=32, p=0.0642) or **(H)** DNMT3B (n=27) but was significantly correlated with **(F)** DNMT1 (n=29, *p<0.05) and **(G)** DNMT3A (n=30, *p<0.05).

We next investigated whether variation in dopamine receptor mRNA expression (DRD1-DRD5) was associated with differences in IL-1β DNA methylation and epigenetic enzyme levels. While no significant correlations were observed between dopamine receptor levels and IL-1β DNA methylation (data not shown), baseline MDM expression of several epigenetic enzymes showed robust positive associations with dopamine receptor expression. Notably, the strongest correlations were observed with D1-like receptors, DRD1 and DRD5. Specifically, TET2, DNMT1, and DNMT3A exhibited significant positive correlations with DRD1, while DNMT3B showed a similar trend (**Figures 6A–D**, TET2 vs DRD1, n = 32, Spearman r = 0.4265, *p = 0.0149; DNMT1 vs DRD1, n = 29, Spearman r = 0.5030, **p = 0.0054; DNMT3A vs DRD1, n =30, Spearman r = 0.5874, ***p = 0.0006; DNMT3B vs DRD1, n =30, Spearman r = 0.3761, p = 0.0532). Comparable positive correlations were also observed for TET2, DNMT1, and DNMT3A with DRD5 (**Figures 6E–G**, TET2 vs DRD5, n = 32, Spearman r = 0.3310, p = 0.0642; DNMT1 vs DRD5, n = 29, Spearman r = 0.3842, *p = 0.0396; DNMT3A vs DRD5, n =30, Spearman r = 0.4287, *p = 0.0181).

**Figure 7:**
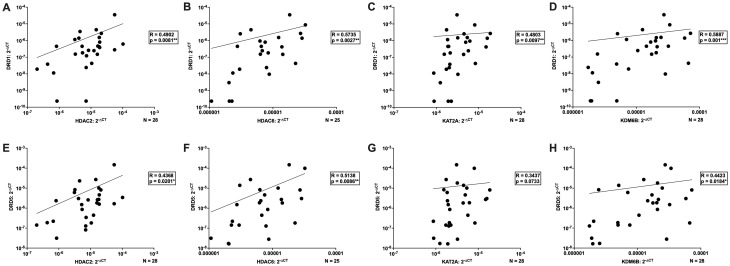
Baseline D1-like dopamine receptor expression correlates with involved in histone acetylation and methylation in primary human macrophages. Donors from **Figure 3** were combined with an independent archival cohort to assess mRNA correlations between dopamine receptor expression and epigenetic enzyme expression. DRD1 expression was significantly correlated with **(A)** HDAC2 (n=28, **p<0.01), **(B)** HDAC6 (n=25, **p<0.01), **(C)** KAT2A (n=28, **p<0.01), and **(D)** KDM6B expression (n=28, ***p<0.001). DRD5 expression was significantly correlated with **(E)** HDAC2 (n=28, *p<0.01), **(F)** HDAC6 (n=25, *p<0.01), and **(H)** KDM6B (n=28, *p<0.05), but not with **(G)** KAT2A expression (n=28, p=0.0733).

For the histone modifying enzymes, HDAC2, HDAC6, KAT2A, and KDM6B all displayed significant positive correlations with DRD1 (**Figures 7A–D**, HDAC2 vs DRD1, n = 28, Spearman r = 0.4902, **p = 0.0081; HDAC6 vs DRD1, n = 25, Spearman r = 0.5735, **p = 0.0027; KAT2A vs DRD1, n = 28, Spearman r = 0.4803, **p = 0.0097; KDM6B vs DRD1, n = 28, Spearman r = 0.5887, ***p = 0.001). Similarly, these enzymes also showed positive correlations with DRD5 (**Figures 7E–H**, HDAC2 vs DRD5, n = 28, Spearman r = 0.4368, *p = 0.0201; HDAC6 vs DRD5, n = 25, Spearman r = 0.5138, **p = 0.0086; KAT2A vs DRD5, n = 28, Spearman r = 0.3437, p = 0.0733; KDM6B vs DRD5, n = 28, Spearman r = 0.4423, *p = 0.0184).

In contrast, few associations were observed for D2-like receptors. Only DNMT1 exhibited a significant positive correlation with DRD3 (DNMT1 vs DRD3, n = 29, Spearman r = 0.4394, *p = 0.0171, data not shown), and DNMT3B showed a trend toward a negative correlation with DRD4 (DNMT3B vs DRD4, n = 27, Spearman r = -0.3557, p = 0.0686, data not shown).

Overall, these findings indicate that epigenetic enzyme expression in macrophages is predominantly associated with D1-like receptor levels. This pattern is consistent with our previous observations that dopamine-mediated regulation of macrophage pro-inflammatory signaling is largely driven by D1-like receptors (68, 97), highlighting a potential mechanistic link between D1-like receptor expression and epigenetic regulation in immune cells. Notably, variability in baseline dopamine receptor expression across donors may establish individual signaling setpoints that constrain the magnitude and direction of epigenetic responses to dopamine.

## 4 Discussion

In this study, we demonstrate that dopamine increases inflammatory gene expression in primary human macrophages through epigenetic mechanisms. Dopamine exposure altered IL-1β expression in a DNA methylation-dependent manner, as pharmacological inhibition of DNMT activity abolished dopamine-induced transcriptional changes. In parallel to changes in methylation, dopamine increased the expression of multiple epigenetic enzymes, including TET2, HDAC2, and HDAC6, which are key regulators of inflammatory gene expression in myeloid cells (87, 92, 93, 98). Together, these results suggest dopamine can act as a novel modulator of inflammatory gene expression through mechanisms previously implicated in classical macrophage activation. Similar epigenetic and inflammatory responses to catecholaminergic signaling have been observed in microglia (99, 100), where neurotransmitter-driven changes in cytokine expression and chromatin remodeling contribute to neuroimmune activation. These parallels suggest that dopamine-mediated epigenetic regulation may represent a conserved mechanism across peripheral macrophages and CNS-resident myeloid cells. While this study focused on pharmacological inhibition of DNA methylation, future studies should investigate whether the TET and HDAC pathways, which showed dopamine-associated transcriptional changes, contribute to dopamine-mediated induction of IL-1β expression.

The requirement for DNMT activity implicates DNA methylation as a key mediator of dopamine-induced inflammatory responses. While promoter methylation is typically associated with transcriptional repression, it can have context-dependent effects. Indeed, although dopamine increases IL-1β promoter DNA methylation, it concurrently increased IL-1β mRNA expression, and we have previously shown it can increase IL-1β production (67, 68). This apparent paradox may reflect the dynamic nature of protein–DNA interactions: methylation can block transcription factor binding directly or via recruitment of methyl-CpG-binding proteins, but it can also create novel binding sites or alter transcription factor sequence specificity (101). Future studies could directly assess dopamine-induced changes in transcription factor binding and chromatin remodeling at key inflammatory loci such as IL-1β using chromatin immunoprecipitation (ChIP)-based approaches, including assessment of associated histone modifications such as H3K27ac and H3K27me3 (102–104). In addition to NF-κB activation and NLRP3 inflammasome recruitment (67, 68), other factors should be explored such as AP-1, which is regulated by DRD1 signaling (105) and whose binding sites are enriched in many genes upregulated by substances of misuse (106), as well as negative regulators like SP1 (107). We previously found that blockade of dopaminergic signaling using the pan-dopamine receptor antagonist flupentixol abrogated IL-1β mRNA expression in microglial cell lines (68). Human primary macrophages and microglia typically express higher levels of D1-like receptors relative to D2-like receptors (59), and inflammatory responses to dopamine are greater in macrophages with a higher D1:D2 receptor ratio. Understanding which transcription factors and epigenetic modifications are recruited or blocked downstream of specific dopamine receptors will be critical for linking epigenetic remodeling to functional changes in inflammatory gene expression.

Although enzyme expression does not directly equate to enzymatic activity, the concurrent dopamine-induced changes in HDAC2 and HDAC6 expression further suggest that dopamine engages dynamic chromatin remodeling processes, potentially coordinating DNA methylation with histone modifications to fine-tune inflammatory gene expression. Given the highly variable kinetics of epigenetic enzyme activity across the genome (108), dopamine-induced modulation of epigenetic enzymes may produce disproportionate, gene-specific effects on inflammatory responses. TET2, for example, may recruit HDAC1/2 to specific inflammatory gene promotors in a DNA methylation-specific manner (85). These dynamic, dopamine-driven epigenetic changes raise the intriguing possibility that dopamine may contribute to trained immunity–like phenomena in macrophages. Trained immunity describes the persistence of epigenetic and metabolic reprogramming following transient stimuli, resulting in altered inflammatory responsiveness upon subsequent challenges (109, 110). Catecholamines have been shown to induce trained immunity in monocytes (44), and our findings suggest that dopamine may similarly trigger lasting epigenetic events capable of modulating macrophage inflammatory setpoints. Beyond IL-1β, we have previously shown that dopamine alters additional cytokines and chemokines, such as IL-6, TNF, CXCL8, CXCL10, CXCL9, and CCL2 (97). Some of these inflammatory mediators likely share transcriptional regulators with IL-1β, whereas others may be controlled through distinct signaling and epigenetic pathways. Future transcriptomic and ATAC-seq analyses could help define cytokine-specific gene regulatory networks and identify chromatin accessibility changes associated with dopamine signaling. By analogy to activity-dependent DNA methylation in neurons, which underlies learning and memory (111, 112), dopamine-mediated epigenetic remodeling in myeloid cells could establish long-term changes in responsiveness to inflammatory cues, particularly in tissues with high dopaminergic tone. While trained immunity was not directly assessed in this study, these results suggest a mechanism by which dopamine may shape the capacity of innate immune cells to respond to future stimuli, with potential implications for neuroinflammation and dopaminergic disorders.

Interestingly, we observed that dopamine-induced epigenetic changes varied with donor sex and age. Males and younger donors (<40 years) generally exhibited greater modulation of epigenetic enzyme expression, including DNMTs and HDACs, compared to females and older donors. These findings suggest that biological variables can influence the sensitivity of macrophages to dopaminergic signaling. Consistent with this, we also observed that baseline dopamine receptor expression correlates with epigenetic enzyme levels, particularly for D1-like receptors (DRD1 and DRD5). Several enzymes, including TET2, DNMT1, DNMT3A, HDAC2, HDAC6, KAT2A, and KDM6B, showed positive associations with DRD1 and, to a lesser extent, DRD5. Although these results are limited by cohort size, they suggest that inter-individual variation due to donor demographics or dopamine receptor expression may contribute to heterogeneity in dopamine-mediated epigenetic and inflammatory responses. Importantly, these findings also underscore that dopamine signaling in macrophages is fundamentally distinct from canonical neuronal dopamine signaling (63, 64). Non-neuronal cells express different receptor repertoires and engage alternative downstream pathways, which likely shape the context-specific epigenetic consequences of dopamine exposure. In this light, the receptor–enzyme correlations observed in macrophages provide mechanistic insight into how peripheral immune cells may interpret dopaminergic cues differently from neurons.

Epigenetic studies of dopaminergic dysregulation have traditionally focused on brain regions implicated in substance use disorders and neuropsychiatric disease. However, limited access to human brain tissue poses a major barrier to mechanistic investigations. Emerging evidence suggests that peripheral immune cells may serve as informative surrogates: stimulant drugs such as cocaine induce coordinated changes in DNA methylation machinery in both brain regions, including the nucleus accumbens, and in peripheral blood leukocytes, with correlated alterations in DNMT and TET activity (41, 51). Our findings extend this framework by demonstrating that dopamine directly reshapes epigenetic programs in human macrophages, supporting a role for peripheral immune cells as both reporters and contributors to dopaminergic and inflammatory processes. Larger, well-characterized cohorts and genome-wide epigenomic profiling of patient-derived immune cells could further uncover dopamine-associated epigenetic signatures across neuropsychiatric and neurodegenerative conditions.

At the same time, accessible human models of the brain are critical for dissecting CNS-specific mechanisms, making brain organoids a valuable direction for future studies. With regards to substance use disorders, drug-induced dopaminergic transmission is known to alter epigenetic pathways, but it remains unclear whether these effects occur directly or via intermediate dopaminergic signaling. Dopamine agonists alone can modulate histone acetylation, DNA promoter methylation, and HDAC activity (6, 31), yet the epigenetic impact of individual substances of misuse in the absence of intrinsic dopaminergic tone remains largely unexplored. Emerging non-animal models, such as iPSC-derived neural systems, including brain organoids cultured with CNS myeloid cells like microglia (113–115), offer a powerful platform to interrogate human-specific molecular responses to addictive substances with or without endogenous dopamine. Coupled with high-resolution single-cell analyses and co-immunoprecipitation studies, these approaches provide the opportunity to dissect nuanced effects of neurotransmitter dysfunction on epigenetic regulation, effectively bridging insights from peripheral immune cells to central nervous system processes.

In conclusion, our study identifies dopamine as an epigenetic regulator of inflammatory gene expression in human macrophages, highlighting a previously underappreciated role in modulating innate immune function. By reshaping myeloid epigenetic programs, dopamine may contribute to the pathophysiology of neuropsychiatric and neurodegenerative disorders marked by both dopaminergic dysfunction and immune activation. Characterizing these myeloid-specific epigenetic mechanisms opens avenues for precise therapeutic strategies: coupled with emerging gene-editing and epigenetic-modifying technologies (116), this knowledge could guide the development of targeted interventions, inform patient stratification, and reveal potential contraindications, positioning dopamine-sensitive immune pathways as a critical nexus for translational research.

## Data Availability

The original contributions presented in the study are included in the article. Further inquiries can be directed to the corresponding author.
